# Advances in the prevention of oral disease; the role of the International Association for Dental Research

**DOI:** 10.1186/1472-6831-15-S1-S8

**Published:** 2015-09-15

**Authors:** Helen Whelton, Christopher Fox

**Affiliations:** 1 School of Dentistry, University of Leeds, Leeds, LS2 9LU, UK; 2 Executive Director, International Association of Dental Research, 1619 Duke Street, Alexandria, VA 22314-3406 USA

## Abstract

**Abstract:**

**Background**

Since its foundation in 1920, prevention of oral disease has been a priority for the International Association for Dental Research (IADR) and the commitment of the organisation to the subject area is clearly expressed in its mission to improve oral health worldwide. The IADR has a current global membership of almost 11,000 people who share an interest in oral and craniofacial research.

**Contribution of IADR**

This paper provides an overview of the contribution of IADR to supporting research and associated activities in disease prevention, in disseminating knowledge and in advocating for better oral health for all citizens of the world. It looks back over time and summarises current supports. Two more recent initiatives in disease prevention are described in more detail, the Global Oral Health Inequalities Research Agenda (GOHIRA) and the proceedings at the 2013 World Conference on Preventive Dentistry (WCPD, 2013), a joint initiative between IADR and WHO.

Through organisational structure, meetings, publications, scientific groups and networks and external relations, IADR has been at the forefront of advancing research for the prevention of oral diseases.

**Conclusions:**

IADR is committed to ensuring research advances get disseminated and implemented and at the same time encourages and advocates for basic, clinical and translational research across disciplines so that we may uncover the major breakthrough in prevention of oral disease.

## Background

### Historical context

Efforts to maintain good oral hygiene are to be found in historical texts. Around 3000 B.C the first known dentist Hesi Re used to promote rinsing with Bicarbonate of Soda to clean the mouth. Hippocrates (460-377 BC) also attributed great importance to cleaning the teeth, he attributed dental disease to “a combination of natural predisposition and the corroding action of accumulated filth". Prior to the development of toothbrushes as we know them now, chewing sticks were used to clean the teeth, Mohammed (570-632) encouraged the use of Miswak (a twig from the Salvadora Persica tree) to clean the teeth before prayer. The use of Miswak dates back 7000 years to the Pharaohs and ancient Babylonians. In more modern times in 1728 Pierre Fauchard recommended treatment of oral infection as follows “rinse out the mouth every morning and also evening with some spoonfuls of your own urine just after it has passed”, fortunately it was one recommendation that did not endure. With the passage of time development of scientific equipment and techniques enabled the study of disease to evolve from empiricism to the application of scientific method and logic to the investigation about its aetiology and prevention. By the 19^th^ century, scientists such as GV Black (1836-1915) were able to study dental pathology and WD Miller (1853-1907) to develop his study of bacteriology.

It was a desire to bring scientific advances to bear on the study of oral diseases and improvement of oral health that led to the founding of the *Journal of Dental Research* (JDR) in 1919 by William J. Gies, and the founding in 1920 of the International Association for Dental Research (IADR). Gies (1872-1956) was a distinguished Biochemist at Columbia University in New York. As the pace of scientific discovery increased in the 20^th^ century the role of printed journals and scientific conferences gained increasing importance for informing researchers and keeping them up to date with advances in dental research. The JDR was initially published quarterly (1919), then every two months (1928) and monthly (1977). Some early articles in JDR included “Prophylaxis” [1], “Results of Five Years of Dental Prophylaxis for Employees of the Metropolitan Life Insurance Company” [2], and “Ideal Tooth Brushing” [3]. Both the Journal and the IADR meetings were central to the communication and review of all aspects of dental research including preventive dentistry. Later in the 20^th^ century, the role of fluoride in preventing dental caries was being uncovered and many landmark discoveries were either presented at IADR meetings and/ or published in the *Journal of Dental Research*. Indeed, the fluoridation of public water supplies is often heralded as one of the most successful public health interventions of the Century.

The century since the inception of IADR has seen an explosion of scientific knowledge and understanding of the mechanisms of disease and its prevention. The information revolution of the last thirty years has spawned an era of instant communication and easy dissemination of knowledge, the association and its journal have kept pace with these developments by bringing JDR online in 2002, then back digitising all content from 1919, and making JDR online a member benefit accessible to all members.

## Methods

### IADR support for advancement of oral disease prevention

**Figure 1:**
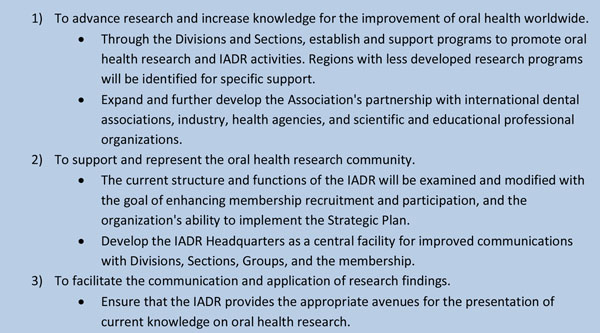
The Mission of the International Association for Dental Research.

The mission of IADR incorporates a clear commitment to the prevention of oral disease and is worth setting out in this context, see Figure 1.

**Figure 2:**
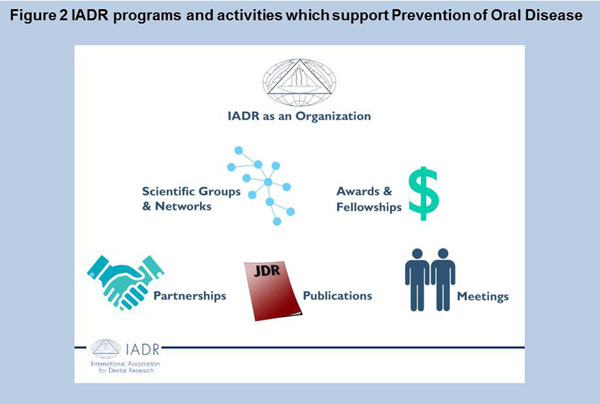
Details of IADR programs and activities, which support and encourage the Prevention of Oral Disease.

Over time the programs and activities to support oral health research have grown in number and diversity, (Figure 2). Support for the advancement of disease prevention has been embedded in these initiatives. In general terms, the creation of groups and networks, of which there are thirty, facilitated communication amongst researchers in areas of common interest. Prevention of disease is embedded within groups and networks where it is relevant. Group and network scientific programs and symposia allow a focus on new and emerging areas and allow those interested in the subject easy access to the latest developments in their area of interest. The meeting program is designed to enable easy identification of topic areas and to allow attendees to create their own schedules in advance, allowing those with a focus on prevention to pursue their research interest and develop their knowledge.

**Figure 3:**
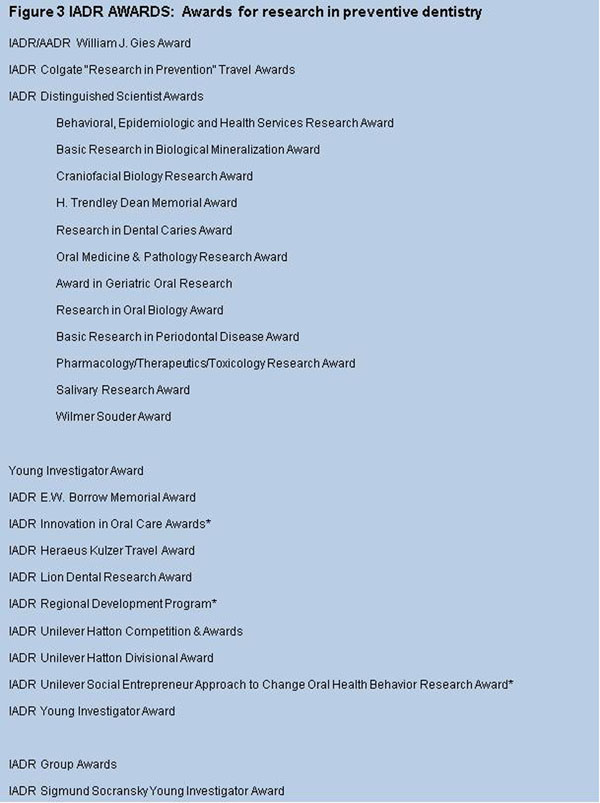
IADR Awards - Details of the specific awards available to researchers pursuing a preventive theme with links to each award description and criteria. Some of the awards are explicitly for research in preventive dentistry either at individual or population level, others less obviously so but do embrace the area. The awards encompass a broad spectrum of disciplines including cariology, periodontology, oral biology, microbiology, immunology, material science, behavioural science, mineralised tissue research or craniofacial growth all of the areas included encompass a potential for focus on prevention of oral disease.

Awards and fellowships are awarded by the overall organisation and also by groups and networks. The specific awards available to researchers pursuing a preventive theme are presented in Figure 3 with links to each award description and criteria. Some of the awards are explicitly for research in preventive dentistry either at individual or population level, others less obviously so but do embrace the area. The awards encompass a broad spectrum of disciplines including cariology, periodontology, oral biology, microbiology, immunology, material science, behavioural science, mineralised tissue research or craniofacial growth all of the areas included include a potential for focus on prevention of oral disease. The value of these awards is that they recognise and celebrate researchers at various stages of their careers. Dedication to the advancement of science in the prevention of oral disease is thus rewarded and encouraged. Three of these awards (marked with *) go beyond reward and recognition and fund innovative research which includes prevention of oral disease.

There are many examples of outstanding researchers in Preventive Dentistry being recognised at early, mid and late career stages by these awards. There are innumerable examples of research development being played out through IADR, from the early work in the 1940s of Michael Buonocore [4] who referenced an even earlier JDR publication by Manly and Hodge [5] in 1939 in his methods. One of Buonocore's students Lou Ripa was an early Hatton Award winner in the Postgraduate category. More recently, JDR published a landmark study on the use of fluoride varnish for preventing early childhood caries [6].

## Results

### IADR meetings

IADR meetings provide a forum for exchange of ideas and for gaining new knowledge and insight into advances in dental research and create a rich context for inspiring new ideas and developing collaborations. The divisional and sectional meetings as well as the general session provide ample opportunity to present one's own work and gain feedback from colleagues.

### The World Congress on Preventive Dentistry (WCPD) 2013

The World Congress on Preventive Dentistry (WCPD) is a joint initiative between the World Health Organisation and IADR, the meeting is held every four years and is held in different parts of the world to communicate the latest research findings that can have a positive impact on dental public health, and to fulfil part of IADR's Mission, “to facilitate the communication and application of research findings.” The most recent WCPD took place in Budapest, Hungary in September 2013, the congress presented a rich and varied program illustrating progress in the prevention of oral disease, it also demonstrated areas where there is potential for future development and creation of new knowledge. The main program heard evidence from global, regional and national programs regarding effective oral disease prevention and community health promotion, approaches to addressing the needs of vulnerable populations, common risk factor and population approaches to disease prevention and the associations and commonalities among oral and systemic diseases. The critical role of health policy was related in a consideration of the integration of health in all policies and its importance in effective governance for health and equity (A. UUTELA, National Institute for Health and Welfare, Helsinki, Finland). A session on new and emerging technologies for Health Promotion heard about Salivary Diagnostics and their use in Primary Healthcare (D.T. WONG, University of California - Los Angeles), Smartphone and mHealth Innovations for Early Diagnosis (P. BIRUR, K.L.E.S’ Institute of Dental Sciences’, Karnataka, India) and Use of Social Media to Promote Oral Health (A. NATTESTAD, University of the Pacific, San Francisco).

Workshops and sponsored symposia are important features of WCPD, an IADR Global Oral Health Inequalities Research Agenda® (IADR-GOHIRA®) workshop was held at the start of the 2013 WCPD meeting. GOHIRA is a key initiative of the IADR Board and it is described along with the symposium following this section.

Two satellite symposia focused on effective approaches to caries prevention, one symposium presented accounts of Implementation Successes (sponsored by Proctor and Gamble) and heard examples of three very different and successful oral health promotion initiatives. The first was Childsmile, the Scottish public sector child oral health improvement programme (L. McPherson, University of Glasgow Dental School); the second was a report on Public-Private Approaches to Oral Health Promotion in Vulnerable Communities (C. A. Evans, University of Illinois at Chicago, College of Dentistry) and the third was on approaches in general dental practice entitled Prevention-Focused Patient Care in the Dental Practice (R Compton, DentaQuest Institute).

The second symposium sponsored by GABA International was titled New Approaches to Caries Prevention and Management. The meeting heard from Nigel Pitts (Kings College, London) about the key objective of The Alliance for a Cavity Free Future (ACFF) which is to bring together all interested stakeholders who support a common purpose to: “STOP CARIES NOW for a cavity-free future". The potential of the Alliance to improve health and also to reduce inequalities were outlined.

Svante Twetman (University of Copenhagen) presented the contemporary case for management of caries through controlling the stability of oral biofilm in all its diversity and complexity rather than targeting specific oral pathogens. He identified and gave examples of four main approaches i) use of metabolic inhibitors (fluoride, xylitol), ii) sugar frequency interventions, iii) saliva stimulation, and iv) novel strategies, such as probiotics, targeted antimicrobial peptides and alkali supplements.

The third presenter, Professor Cor van Loveren (ACTA, Amsterdam), spoke about ‘Stagnation in prevention, where do we go?’ He illustrated with examples a number of ways to successfully breach the stagnation in prevention. He cited examples of different approaches along the upstream downstream path: the success of the Childsmile intervention in Scotland as a community based interventions (http://www.child-smile.org.uk); the development of novel anti-caries and remineralising agents (Advances in Dental Research 2012;24;#2); the success across the social class spectrum of a non-operative caries treatment program (the Nexø-model) which is a dental health care program based on individualised non-operative caries treatment of children and adolescents. Importantly he stressed the need to incorporate pay for performance in health care systems to remunerate and encourage prevention.

While the WCPD provides a focused forum for preventive dentistry every four years, the regular divisional, regional and annual general session of IADR provide ongoing opportunities to present and learn about advances in preventive dentistry.

### Global oral health inequalities research agenda IADR-GOHIRA®

IADR-GOHIRA® is an initiative of IADR which aims to articulate a research agenda which will ultimately generate evidence to inform strategies to successfully address inequalities in oral health, [7], [8], [9]. IADR recognises that addressing this challenge will require closer and more robust engagement across sectors, including social policy, and the adoption of an upstream approach that integrates action on oral health with approaches to reduce the global burden of non-communicable disease in general. IADR has published a call to action[9] to focus the attention of international leaders in oral health research on this issue. This initiative illustrates the leadership role of IADR in promoting disease prevention across all sectors and geographic areas.

The WCPD IADR-GOHIRA® workshop focused on what actions were necessary to address:

• gaps in knowledge and specifically insufficient focus on translational research and social policy;

• integrating oral health strategies with general health;

• inadequate evidence-based data, including: research drivers.

The workshop combined interactive round table discussions with presentations on The Global Burden of Disease Study 2010.; “What this tells us about oral health inequalities” (W Marcenes), “Mainstreaming oral health: upstream, downstream and midstream” (R. Watt) and “Learning from effective upstream interventions: the lessons of strong legislation in the fight against obesity (J. Chriqui)".

**Figure 4:**
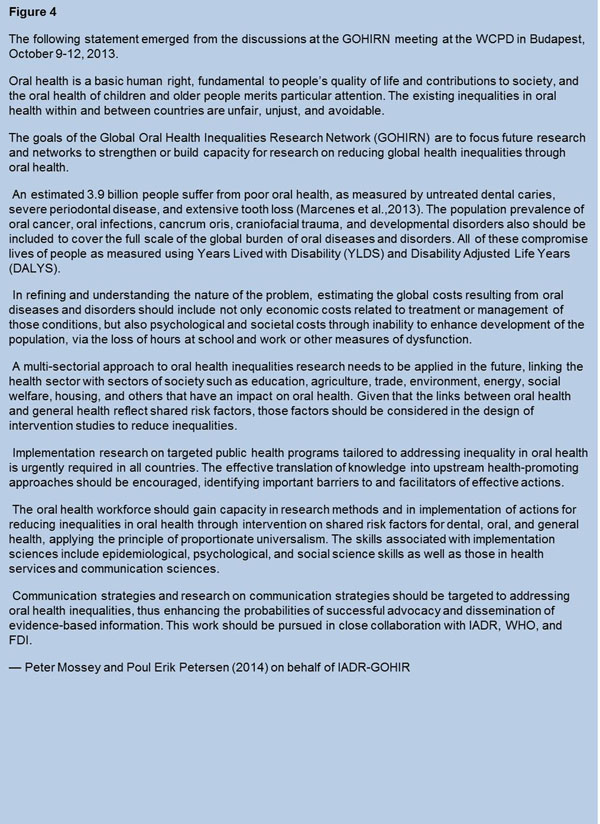
Budapest Declaration, A statement drafted following an interactive workshop hosted by the Global Oral Health Inequalities Network of the International Association for Dental Research at The World Congress on Preventive Dentistry in Budapest, Hungary in September 2013.

Following the workshop the group drafted “The Budapest declaration” [10] (Figure 4).

### The Journal of Dental Research (JDR)

Throughout the history of IADR, the *Journal of Dental Research* has supported the mission of the organisation through publishing high quality dental research, the impact of JDR is clear with an impact factor of 3.826, the second highest of all 83 dental journals. The recent publication of the Clinical supplements, periodic publication of *Advances in Dental Research* and the dissemination of the Global Research Update newsletter further support the dissemination of research including that in prevention of oral diseases. Most recently IADR has added the use of PodCasts to its publication strategy with the launch of its first PodCast. In this podcast we hear Harold Slavkin speaking about his JDR paper [11] From Phenotype to Genotype, Enter Genomics and Transformation of Primary Health Care around the World. [12]. Dr Slavkin heralds “a new era using human and microbial whole-genome sequencing to make significant healthcare decisions as to risk, stratification of patients, diagnosis, treatments, and outcomes". He challenges us to “invest in genomics to enhance clinical oral health care in the 21st century for all people"; he also encourages us to revisit Interprofessional Education and health teams (IPE) with the goal of improving “the depth, breadth, and quality of comprehensive and coordinated health care across the lifespan."

### Worldwide participation

From its inception, the IADR has always been international. Initially this was more in name than actual practice, but the globalisation of IADR has increased rapidly, particularly in the last decades. The founding members of IADR in 1920 were all from Boston, New York, and Chicago. The first IADR meeting outside of the United States was in Toronto in 1930 and the first meeting outside North America was not until 1975 in London. More recently, between 2006 and 2016, the IADR General Session will have been on every habitable continent in the world. Membership has gone from 100% U.S. in 1920 to just about 1/3 in 2013. The early international membership growth came from Canada and Europe, but more recently from Latin America and Asia. In 2007, the IADR instituted a tiered-dues structure, which provided a much reduced dues for members residing in World-bank classified low and middle-income countries. The potential for further growth outside North America and Europe is enormous and will further allow IADR to bring a message of oral health research and prevention.

### Global partnership for prevention

Beyond Scientific Groups and Networks, Awards and Grants, Meetings and Support for the Journal of Dental Research, External Partnerships and representing the dental community on Global Dental Affairs is a key role for IADR. The World Health Organisation (WHO) has just two non-governmental organisations with a dental focus in official relations, the IADR and the FDI World Dental Federation. There have been frequent collaborations between WHO, the FDI and IADR, including, but not limited to, the Global Goals for Oral Health Policy Statement, adopted in 2003, a series of Fluoride meetings in Geneva, Beijing, and Thailand, and joint cooperation on scientific and technical support for the United Nations Environmental Programme International Negotiations Committee leading to the Minamata Convention on Mercury.

## Conclusions

In summary, through our organisational structure, our meetings, publications, scientific groups and networks and our external relations, IADR has been at the forefront of advancing research for the prevention of oral diseases. IADR is committed to ensuring these research advances get disseminated and implemented and at the same time encourage and advocate for basic, clinical and translational research across disciplines so that we may uncover the “next fluoride".

## Competing interests

Helen Whelton received partial funding from Colgate Palmolive to attend the Prevention in Practice Conference. Christopher Fox is the Executive Director of IADR.

## Authors’ contributions

Both authors have made substantial contributions to conception and design, have been involved in drafting the manuscript and revising it critically for important intellectual content; have given final approval of the version to be published and agree to be accountable for all aspects of the work in ensuring that questions related to the accuracy or integrity of any part of the work are appropriately investigated and resolved.

## Declarations

This article has been published as part of *BMC Health Services Research* Volume 15 Supplement 1, 2015: Proceedings of Prevention in practice - making it happen. The full contents of the supplement are available online at http://www.biomedcentral.com/bmchealthservres/supplements/15/S1. Publication charges for this supplement were funded by Colgate Palmolive.
